# Impaired social decision making in patients with major depressive disorder

**DOI:** 10.1186/1471-244X-14-18

**Published:** 2014-01-23

**Authors:** Yun Wang, Yuan Zhou, Shu Li, Peng Wang, Guo-Wei Wu, Zhe-Ning Liu

**Affiliations:** 1 Key Laboratory of Behavioral Science, Institute of Psychology, Chinese Academy of Sciences, Beijing 100101, China; 2 University of Chinese Academy of Sciences, Beijing 100049, China; 3 Institute of Mental Health, Second Xiangya Hospital of Central South University, Changsha 410011, China

**Keywords:** Ultimatum game, Major depressive disorder, Decision making, Fairness

## Abstract

**Background:**

Abnormal decision-making processes have been observed in patients with major depressive disorder (MDD). However, it is unresolved whether MDD patients show abnormalities in decision making in a social interaction context, in which decisions have actual influences on both the self-interests of the decision makers *per se* and those of their partners.

**Methods:**

Using a well-studied ultimatum game (UG), which is frequently used to investigate social interaction behavior, we examined whether MDD can be associated with abnormalities in social decision-making behavior by comparing the acceptance rates of MDD patients (N = 14) with those of normal controls (N = 19).

**Results:**

The acceptance rates of the patients were lower than those of the normal controls. Additionally, unfair proposals were accepted at similar rates from computer partners and human partners in the MDD patients, unlike the acceptance rates in the normal controls, who were able to discriminatively treat unfair proposals from computer partners and human partners.

**Conclusions:**

Depressed patients show abnormal decision-making behavior in a social interaction context. Several possible explanations, such as increased sensitivity to fairness, negative emotional state and disturbed affective cognition, have been proposed to account for the abnormal social decision-making behavior in patients with MDD. This aberrant social decision-making behavior may provide a new perspective in the search to find biomarkers for the diagnosis and prognosis of MDD.

## Background

Major depressive disorder (MDD) is characterized by a persistent and overwhelming feeling of sadness [1]. Emotional processes, including emotional response and feeling states, are an important factor with a prominent impact on decision making [2,3]. Previous studies have found that the abnormal feeling state in patients with MDD may bias their decision-making behaviors, as evidenced from altered sensitivity to reward and punishment [4-7], reduced experiences of regret [8], and poor decision performance [9]. Despite these important findings in MDD, all these studies have investigated people in non-social interaction contexts, in which actions only have consequences for the self-interests of the participants [4-8]. However, many real-life decision problems involve social exchanges with other individuals and a certain division of economic outcomes among them [10]. The influence of the depressive state on real-life economic decisions has rarely been studied in a social interaction context. Considering that MDD affects up to 20% of the worldwide population [11,12], the influence of the depressive state on social-economic decisions is an important research question. Ecologically valid social decision-making paradigms such as the ultimatum game (UG) may help identify suboptimal choices associated with MDD and, thus, may provide a potential bridge for translation research in MDD [13-15].

The UG is a commonly used paradigm to study the process of decision-making in a social interaction context [16]. In the UG, a proposer suggests a way to divide a fixed sum of money. The responder has to accept or reject the proposal. If the responder accepts the proposal, the suggested split is realized. If the responder rejects the offer, neither of the two receives anything. In the UG, the payoff-maximizing strategy for the responder (individual level) is to accept all offers, and, reciprocally, for the proposer it is to make the smallest possible offer [16]. However, behavioral experiments from different countries with different stake sizes and different experimental designs confirm that people do not always pursue their own maximum profit [17]. Empirically, approximately half of all unfair offers (defined as approximately 20% or less of the pot) are typically rejected, with increasing rejection rates as offers become less fair [18].

Convergent evidence from psychophysiological measures of emotional arousal [19], behavioral studies on emotional regulation [20-22] and neuroimaging studies [2,23] have documented the role of negative emotion induced by unfair proposals in explaining this rejection behavior observed in the UG. Recent studies have found that temporally changing the baseline emotion or baseline biological states of responders also influences the ultimatum bargaining behavior [24-26]. For example, one recent study found that inducing incidental sadness decreased the acceptance rates of unfair offers in the UG [24]. Other studies found that taking medications that decreased or enhanced serotonin levels could make healthy subjects less or more likely to accept unfair offers, respectively [25,26]. In addition, unfair offers elicited activity in brain areas related to emotion, such as insula and amygdala [2,23]. All these lines of evidence elucidated the role of emotion in ultimatum bargaining behavior. Therefore, we hypothesized that administering the UG task to MDD patients could reveal deviations in their social interactions from those of a healthy population based on the following knowledge about MDD: (1) MDD is characterized by persistent sadness [1]; (2) serotonergic neurotransmission dysfunction is the major pathophysiological hypothesis of this disorder [27,28]; and (3) structural and functional abnormalities in negative emotion-related brain areas, such as the insula and amygdala, have been found in MDD [29,30].

To our knowledge, four studies have addressed this issue, but the results were inconsistent [13,31-33]. Agay et al. (2008) used a two-stage UG paradigm, in which participants played two rounds with the same anonymous opponents, to compare the bargaining behavior of schizophrenic patients with that of MDD patients and of a healthy control group. They found that the acceptance rate of the MDD group was significantly lower than that of the normal control group in the first round and numerically lower in the second round [31]. Using a single-shot UG paradigm, Harlé et al. (2010) found that although depressed individuals reported a more negative emotional reaction to unfair offers, they accepted significantly more of these offers than did the controls [32]. In addition, Destoop et al. (2012) found no significant difference in acceptance rate between severe MDD patients and their healthy controls, although their patients showed a numerically lower acceptance rate [33]. However, in a recent study Scheele et al. (2013) found that compared with healthy controls depressed patients rejected significantly more moderately unfair offers in the UG and rated emotional stimuli as more negative [13]. Therefore, how patients with MDD treat unfair proposals in the UG is still unresolved.

Additionally, no previous studies have investigated whether patients with MDD discriminate between unfair proposals from human partners or computer partners. Studies conducted in healthy populations have found that healthy participants often reject an unfair offer from a human partner but tend to accept the same unfair offer from a computer partner [19,23]. The discriminative responses for unfair proposals from computer partners and human partners argue against the equity model, which holds that subjects’ sense of fairness only depends on whether the distribution is equal [34,35], but they provide a strong evidence for the reciprocity model [36,37]. The reciprocity model posits that a strong reciprocator will punish norm violation behavior to consolidate fairness norms in the social group [36,37], but when the distributor is a computer, the social quality of the interaction is missing, and thus, the reciprocity effect disappears [38]. This indicates that decisions in the UG depend not only on material gains but also on considerations of other agents’ outcomes and intentions [39]. This view has been supported by neuroimaging evidence, which suggests that successful discrimination in responses regarding unfair proposals from computer partners and human partners depends on the intact social cognitive ability, especially the ability to understand and respond to the thoughts and feelings of others [40]. Therefore, a paradigm that includes both human and computer partners may be helpful to improve our understanding of impaired social cognition in patients with MDD. However, in previous MDD studies the participants only played the game with human partners, not with computer partners, leaving unresolved the problem of whether MDD patients can discriminate between unfair proposals from human partners and computer partners.

Furthermore, the offer size changed and the total amount of money to be divided was kept constant in previous UG paradigms, so this type of experimental design mixed fairness and monetary reward. To control for monetary reward, we planned to fix the value of the offer size and to vary the stake size across trials. By keeping the offer size stationary, we can isolate effects of fairness and the size of the offer separately.

The goal of the present study was to examine whether MDD can be associated with abnormalities in decision-making behavior in a social interaction context by comparing the acceptance rate of a group of patients with MDD with that of healthy controls in a UG task in which both human and computer offers were included. We mainly investigated the decision characteristics of MDD patients from the following two aspects: (1) whether there was a difference in acceptance rates between patients with MDD and normal controls in the UG task and (2) whether patients with MDD could treat unfair proposals offered by human and computer partners discriminatively.

## Methods

### Ethics statement

This study was approved by both the Institutional Review Board of the Institute of Psychology, Chinese Academy of Sciences, and the Medical Research Ethics Committee of the Second Xiangya Hospital. All participants gave written informed consent.

### Participants

Fourteen patients were recruited from the Institute of Mental Health, Second Xiangya Hospital of Central South University. The patients satisfied the DSM-IV criteria for a major depressive episode, as diagnosed independently by two qualified psychiatrists who interviewed the patients personally. The psychiatrists had received DSM-IV training for the diagnosis of mental disorders and passed the assessment of consistency prior to the implementation of this study. The patients were excluded if they had any preexisting or concurrent co-morbid primary diagnosis that met the DSM-IV criteria for any Axis I disorder other than MDD. Additional exclusion criteria were acutely suicidal or homicidal behavior, family history of major psychiatric or neurological illness in first degree relatives, history of trauma resulting in loss of consciousness, history of major neurological or physical disorders that could lead to an altered mental state, or current pregnancy or breastfeeding. All the clinical participants were inpatients, except one who was an outpatient. Four of the participants were patients with first-onset MDD, and others were in the relapse phase. The mean frequency of episodes was 1.43 (*SD* = 0.65) times. The mean age of onset was 28.8 (*SD* = 15.5) years old. The mean duration of illness was 46.1 (*SD* = 55.7) months. In the MDD group, only one patient did not take any psychotropic medication; the other patients received antidepressant medications (SSRIs and/or SNRIs), with four patients additionally taking low-dose benzodiazepines and one patient receiving neuroleptics. Nineteen healthy participants were recruited via advertisement as a control group. The control subjects were free of any known psychiatric condition and had never taken any form of antidepressant medication, as screened by a self-reporting questionnaire. Additional exclusion criteria adopted for the normal controls (NC) were the same as those for the MDD group. Immediately before the UG task, the depressive symptoms of the participants were rated by an experienced research physician using the 24-item Hamilton Depression Rating Scale (HDRS; [41]). All participants took part in this study after signing an informed consent form.

### Procedures

#### Cognitive assessment

Decision making encompasses a complex set of processes that requires various higher-order cognitive functions [42]. Previous studies have shown that patients with MDD have impaired cognitive function, although the conclusions are inconsistent [43]. To exclude the potential impact of cognitive dysfunction on decision-making behavior in MDD, a cognitive assessment of all subjects was conducted before the UG task. The Digit Symbol and Information subtests of the Wechsler Adult Intelligence Scale-Chinese Revision (WAIS-RC) were used. The Digit Symbol subtest requires that subjects write down symbols that correspond with figures one to nine in 90 seconds, which reflects a person’s memory and speed of processing. The Information subtest requires participants to answer some common questions, which reflects a person’s range of general information. A similar cognitive assessment was performed in a previous study [13].

#### Ultimatum game

**Figure 1:**
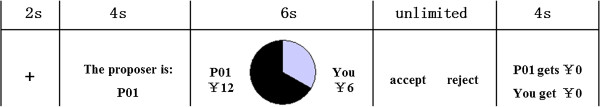
**Diagram illustrating the structure of a single round of the ultimatum game.** Each round began with a 2 s preparation interval. The participant then saw the number of the proposer (e.g., P01) or computer for 4 s. Next, a pie indicating the offer proposed by the partner was displayed for a further 6 s. The participant was given the choice to respond by pressing one button to accept and another to reject the offer with no time limit for this process. Then the result of the choice showed for 4 s.

The UG task was conducted individually, so participants were unable to discuss the research experience with each other. The participants first received instructions explaining the rules of the game, and each participant was required to complete a series of test questions after reading the instructions to verify their comprehension. In the formal experiment, the participants acted as responders in a series of trials of the UG, during which they might play with a computer or with a person. Each trial had 5 phases (Figure 1). The participants were informed that proposals from real persons had been submitted by previous participants and that in each trial their partners would be different (a one-shot game). They were also told that the proposals from the computer were randomly generated. In reality, all the offers were pre-set by the experimenter. In addition, they were told that we would also submit their proposals after the experiment and that their proposals might be adopted in future plays of the game. In reality, the participants’ proposals were not used beyond their function as a cover story. Moreover, to avoid uncontrolled associations, the human proposers were represented by alphanumerical codes, not by their pictures or by real names. Previous studies have found that the decisions of responders in the UG can be influenced by the features of the proposer, such as his/her physical attractiveness [44] and facial emotion [45]. The type of presentation of human proposers we used had been successfully used in a previous UG study [46].

Each responder received 36 offers, 18 of which were supposedly from playing the game with human partners and 18 with computer partners. The offers from the computer partners were identical to those from the human partners, and the rounds were presented randomly. To keep the monetary reward constant and avoid making participants feel bored, we independently manipulated fairness and basic monetary reward (offer size) by varying both the offer amount and the stake size across the trials (Table 1). Two offer sizes (ұ10, ұ4) were proffered, and the offers fell into one of three “fairness” categories: 50%-40% of the stake (fair), 33%-25% of the stake (unfair), or 20%-10% of the stake (most unfair). Thus, there are six combinations of offer size and fairness in each proposer condition, and 3 rounds were set for each combination. Thus, we had 18 trials in each proposer condition. The number of rounds in each fairness condition is comparable with several previous studies [23,47]. In different trials, the same offer amount could represent a larger percentage of the total stake and could, therefore, seem fair, or it could represent a smaller percentage of the total amount and could, therefore, seem unfair. This design controlled for any effects of offer magnitude. Thus, this study was a 2 × 2 × 2 × 3 design, with diagnosis (MDD, NC) as a between-subjects factor and proposer (human, computer), offer size (ұ10, ұ4) and fairness (50%-40%, 33%-25%, 20%-10%) as within-subjects factors. We implemented a random payment method in our experiment. Participants were informed that, after the task, they would be paid in cash based on two randomly chosen offers out of all the proposals.

**Table 1:** Types of offers

	**Fair**	**Unfair**	**Most Unfair**
	**(50%-40%)**	**(33%-25%)**	**(20%-10%)**
**High (ұ)**	10	10	10
	out of 20, 22, 25	out of 30, 35, 40	out of 50, 70, 100
**Low (ұ)**	4	4	4
	out of 8, 9, 10	out of 12, 14, 16	out of 20, 24, 40

#### Questionnaire

After completing the UG task, the participants rated the fairness of all offers presented in the UG task on a Likert scale of 1 (very unfair) to 7 (very fair). The goal of this task was to ensure that the participants’ fairness evaluation criterion was consistent with our classification standards. Then, to increase the degree of their involvement in this experimental situation, they also made offers as proposers with different stake sizes, and we told them their proposals would be used in the subsequent study.

### Statistical analysis

For the analysis of the sample characteristics and the cognitive tests, Student’s t-tests were performed to compare means, whereas chi-square tests were used to compare frequencies.

A 2 (diagnosis: MDD, NC) × 3 (fairness: 50%-40%, 33%-25%, 20%-10%) repeated measures ANOVA was used to test the differences of fairness judgment between the two groups. To compare the differences in acceptance rates, a 2 (diagnosis: MDD, NC) × 3 (fairness: 50%-40%, 33%-25%, 20%-10%) × 2 (proposer: human, computer) × 2 (offer size: ұ10, ұ4) repeated measures ANOVA was conducted. The assumption of sphericity was assessed with Mauchly’s test. Partial eta-squared and Cohen’s *d* were calculated as measures of effect size. When significant effects were found, we conducted post-hoc pairwise Least Significant Difference (LSD) tests.

A partial correlation controlling for age, gender and years of education was used to explore the association between the acceptance rate and depressive severity as indexed by the HDRS scores in the patient group. All reported *p* values are two-tailed or one-tailed if a prior hypothesis regarding the direction of effects was established. Values of *p* < .05 were considered significant. The statistical analyses were conducted using SPSS 17.0.

## Results

### Demographic and clinical data

The major depressive disorder patients (MDD) and normal controls (NC) did not show significant differences in terms of gender composition, age, and educational level (all *p* > .05). The mean HDRS score showed a highly significant difference between the two groups (*p* < .001). No significant differences were found between the groups in their performance on the Digit Symbol and Information portions of the WAIS-RC (both *p* > .05), suggesting that our MDD patients did not show significant cognitive dysfunction in memory, speed of processing, or common sense (Table 2).

**Table 2:** Demographic and clinical details

**Characteristic**	**MDD group**	**NC group**		
	**(*n*** **= 14)**	**(*n*** **= 19)**	***T /χ***^***2 *** **a**^	***p***^**a**^
	**Mean ( *SD *)**	**Mean ( *SD *)**		
Age	32.6 (16.1)	32.9(14.1)	−0.061	0.952
Gender (male:female ratio)	6 : 8	9 : 10	0.066	0.797
Years of education	12.9 (2.5)	14.2 (3.4)	−1.254	0.219
HDRS	21.0 (1.1)	4.6 (1.7)	31.071	<0.001
WAIS-RC: Digital Symbol	57.1 (7.9)	60.1(13.3)	−0.819	0.419
WAIS-RC: Information	16.8 (4.9)	17.7 (3.5)	−0.571	0.572

WAIS-RC: Wechsler Intelligence Scale for Adult-Chinese Revised.

HDRS: Hamilton Depression Rating Scale.

^a^Results of *t*-test and χ^2^ statistics and corresponding levels of significance (two-tailed).

### Fairness judgment

**Figure 2:**
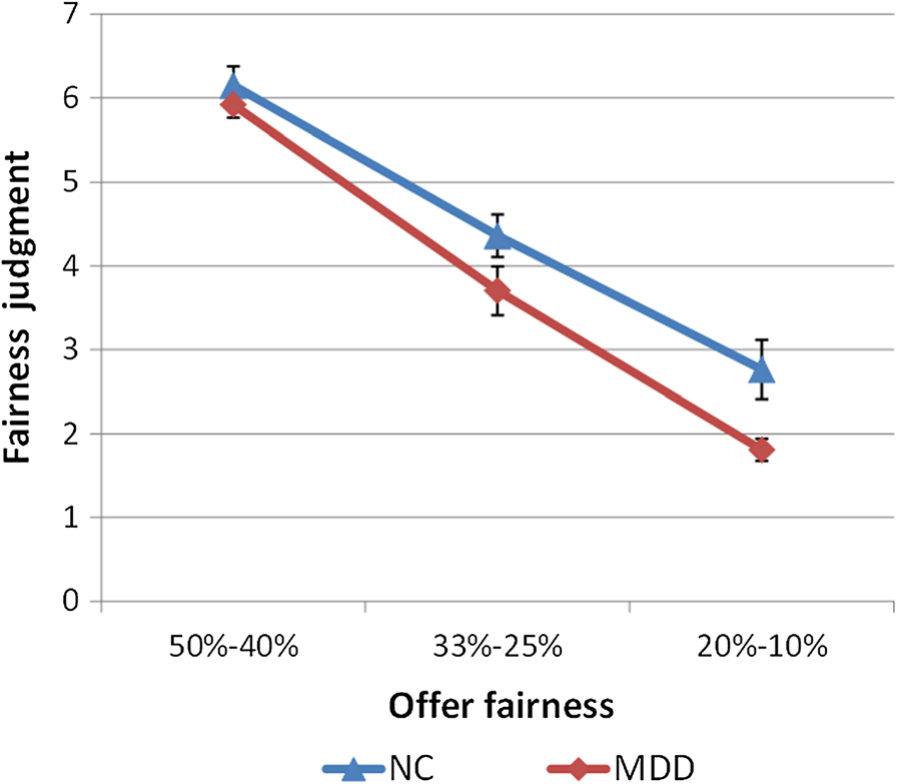
**Comparison of scores on the fairness ratings in the ultimatum game.** Error bars represent the SE of the difference of the means.

To confirm our division of the fairness of each offer and to test for potential differences in justice sensitivity, a repeated measures ANOVA, considering the diagnosis and fairness categories, was conducted. Mauchly’s Test of Sphericity showed the sphericity assumption was met (*p* = .163). The results showed a significant main effect of the fairness category on the participants’ ratings of the fairness of the offers (*F*(2,62) = 195.78, *p* < .001, partial *η*^2^ = .863; all possible pairwise comparisons were significant, all *p* < .001), indicating that the fairness assessment standards of the subjects were consistent with our division. In addition, the main effect of diagnosis was marginally significant (*F*(1,31) = 4.11, *p* = .051, partial *η*^2^ = .117). Because we were more concerned about the group difference in fairness judgment of unfair and most unfair offers, we conducted subsequent independent sample t-tests. The results showed a marginally significant group difference in fairness ratings of unfair offers (MDD: *M* = 3.70, *SD* = 1.09; NC: *M* = 4.36, *SD* = 1.10; *t*(31) = −1.70, *p* = .099, Cohen’s *d* = .602) and a significant group difference in fairness ratings of most unfair offers (MDD: *M* = 1.81, *SD* = 0.50; NC: *M* = 2.76, *SD* = 1.53; *t*(22.89) = −2.54, *p* = .018, Cohen’s *d* = .785) (Figure 2). These results showed that although the patients could also differentiate fairness levels from different proposals, they demonstrated greater sensitivity to unfairness.

### Acceptance rates of UG

**Figure 3:**
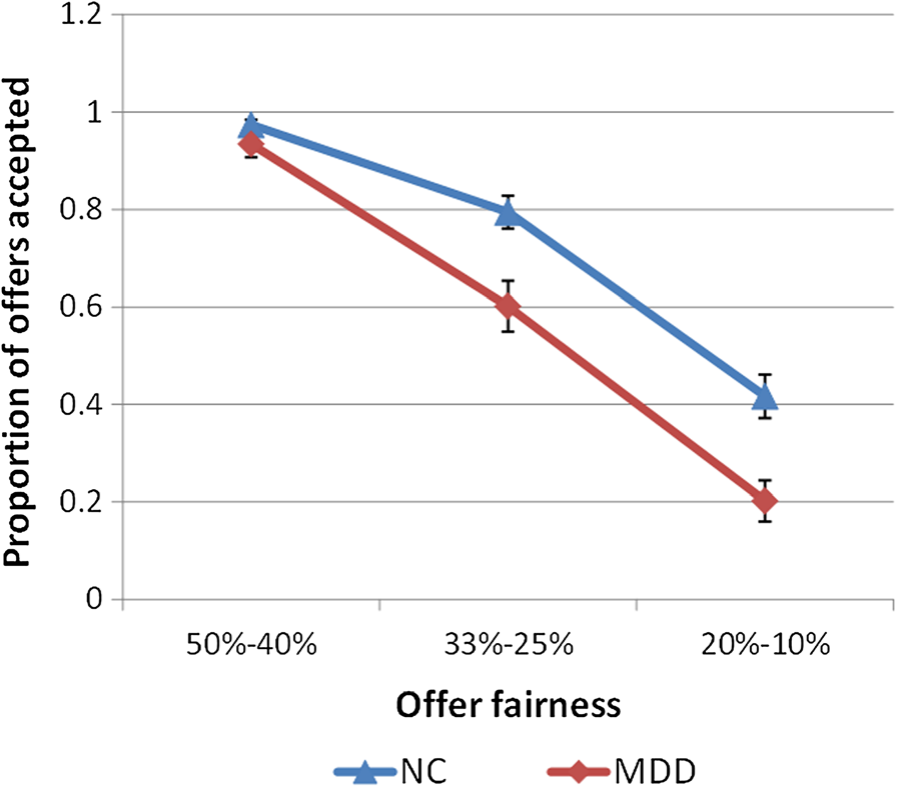
**Effects of offer fairness on acceptance rates by each group in the ultimatum game.** Error bars represent the SE of the difference of the means.

The acceptance rate was investigated by a repeated measures ANOVA, exploring the main effects of diagnosis, fairness, proposer type, offer size and the interaction effects between these factors. Mauchly’s Test of Sphericity showed that the sphericity assumption was met (all *p* > .05). Significant main effects of fairness (*F*(2,62) = 87.18, *p* < .001, partial *η*^2^ = .738; all possible pairwise comparisons were significant, all *p* < .001), offer size (*F*(1,31) = 6.97, *p* = .013, partial *η*^2^ = .184) and diagnosis (*F*(1,31) = 4.87, *p* = .035, partial *η*^2^ = .136) were found. Thus, the acceptance rate decreased when offers became less fair and the acceptance rate was lower facing a lower monetary payoff than facing a higher payoff. In addition, the acceptance rate was lower in MDD than in NC. We did not find any significant interaction effects. Because we were more concerned about the group difference in acceptance rates of unfair and most unfair proposals, we conducted subsequent independent sample t-tests. The results showed a marginally significant group difference in acceptance rates of unfair proposals (MDD: 60%, NC: 79%; *t*(31) = −1.89, *p* = .068, Cohen’s *d* = .662) and a significant group difference in acceptance rates of most unfair proposals (MDD: 20%, NC: 42%; *t*(31) = −2.06, *p* = .048, Cohen’s *d* = .725) (Figure 3). The MDD patients exhibited abnormal decision-making characteristics, evident in the lower-than-normal acceptance rates of the unfair proposals.

**Figure 4:**
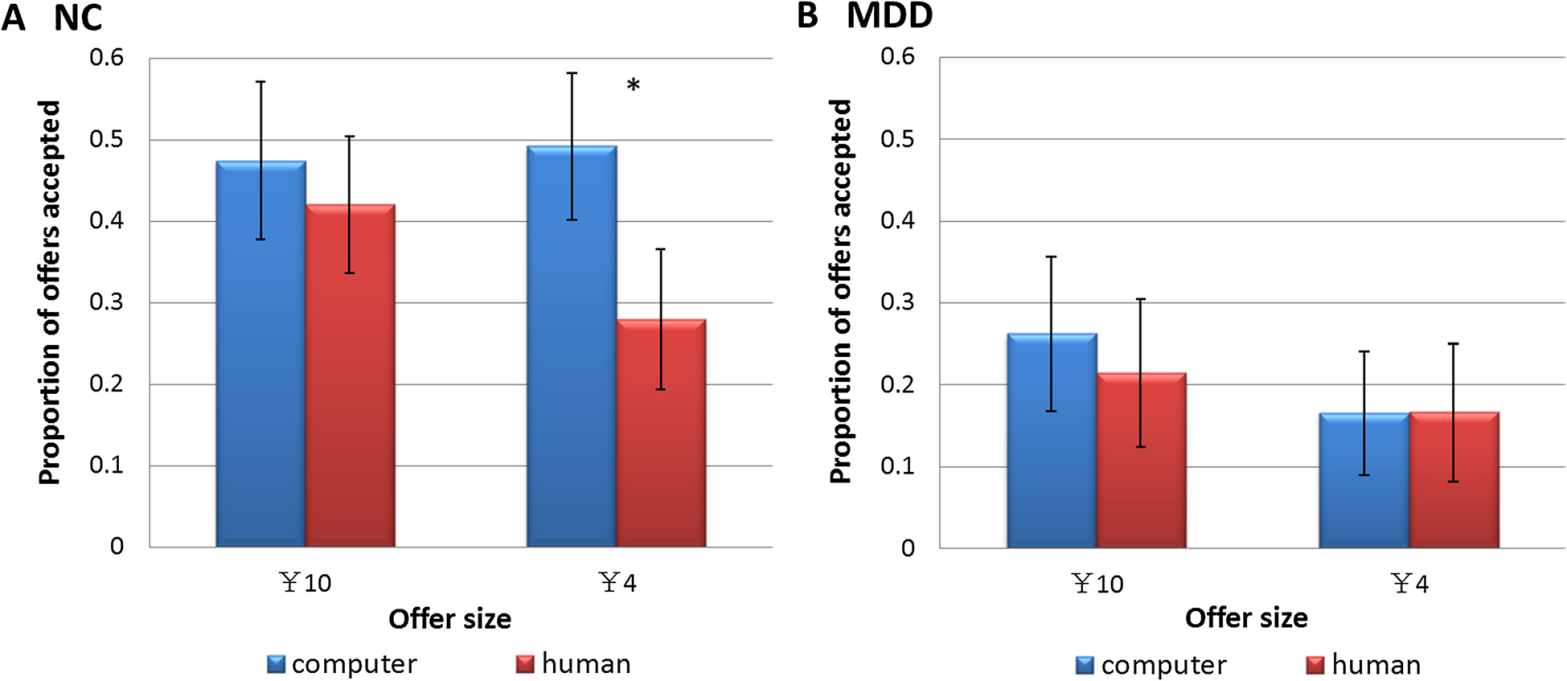
**Behavioral results of normal controls (A) and patients with major depressive disorder (B) to the most unfair offers.** Acceptance rates (%) plotted as a function of offer sizes for different proposers. Error bars represent the SE of the difference of the means. *p < .05.

Previous studies have found that unfair offers made by human partners were accepted at a significantly lower rate than the same offers made by a computer [19,23]. To validate and explore the proposer effect in each group, a paired sample t-test of the proposers was conducted on the unfair and most unfair offer levels to evaluate the offer sizes separately in light of the significant effect of offer size on acceptance rate. The most unfair offers made by the human partners were accepted at a significantly lower rate than the same offers made by the computer partners in the NC group when the offer size was smaller (*t*(18) = 2.73, *p* = .014, Cohen’s *d* = .555). However, no differences between human and computer partners were found in the MDD group (*t*(13) = −0.01, *p* = .992, Cohen’s *d* = .002). When the offer size was larger, no significant proposer effect was found in either group at the most unfair offer level (Figure 4). At the unfair offer level, we did not find any significant effect for proposer type in either group at either offer size.

Comparing the total monetary amount of all offers accepted in the study groups using an independent sample t-test, we found that the subjects in the depressed group earned less money (*M* = 149.43, *SD* = 54.21) than those in the control group (*M* = 185.68, *SD* = 43.42) and that the difference reached significance (*t*(31) = −2.13, *p* = .041, Cohen’s *d* = .751). For the response time in the UG task, an independent sample t-test showed that there was no significant difference in response time between the two groups (*t*(31) = −1.02, *p* = .315, Cohen’s *d* = .360).

### Clinical correlates analysis

The correlation between the acceptance rates and symptom severity, as indexed by the HDRS scores in the patient group, was analyzed by partial correlations with age, gender and years of education as control variables. In general, a significant negative relationship was found between the patients’ acceptance rates and their clinical severity of depression (*ρ* = −0.589, *p* = .028, one-tailed). Moreover, we did not find any significant correlations between the acceptance rates and other clinical features, such as the duration of illness or age of onset.

## Discussion

The goal of this study was to examine whether patients with major depressive disorder show abnormalities in decision-making behavior in a social interaction context, in which their decisions influence both the self-interests of the decision makers *per se* and those of their partners [48], by investigating their acceptance rates in the UG. Our results showed that (1) the acceptance rates of the patients were lower than those of the normal controls and (2) no differences in acceptance rates to unfair proposals were observed in the MDD group while comparing their response to offers from humans with those from computers. Taken together, our findings indicated that the MDD patients showed impaired bargaining behavior when playing the UG.

Previous studies have observed mixed findings in the social decision-making behavior of depressed patients in the UG task. Both decreased and increased acceptance rates by depressed patients have been reported [13,31-33]. Our study supported a decreased acceptance rate of unfair proposals by MDD patients. We speculated that the inconsistency in the ultimatum bargaining behavior of MDD patients may be due to sample heterogeneity. In the only study that reported higher acceptance rates in a depressed group, the participants were recruited from an undergraduate student sample and were typically young, unmedicated patients, and 4 out of 15 had subthreshold MDD [32]. In our study and the other three studies [13,31,33] that reported statistically or numerically decreased acceptance rates in depressed groups, the patients were from a purely clinical population, and most of the patients were taking antidepressant medications. The mixed findings suggest a discrepancy in severity of the disorder (moderate versus more severe forms of depression). Consistent with this speculation, we found a significant negative relationship between patients’ acceptance rates and their clinical severity of depression. This suggests that the severity of depression influences the ultimatum bargaining behavior of MDD patients. Additionally, the mixed findings suggest a role of antidepressant medication in the ultimatum bargaining behavior in MDD patients. A single-dose antidepressant has been found to increase acceptance rates in healthy participants [26]. Along this line, it seems possible that antidepressant medication will increase acceptance rates, yet antidepressants may have a different impact on the brain of depressed patients than on the brain of healthy controls. Long-term antidepressant effects on ultimatum bargaining behavior with treatment-naïve MDD patients should be studied in the future.

Several possibilities have the potential to explain the decreased acceptance rate observed in our MDD patients. First, MDD patients may perceive fairness differently from others. When judging the fairness of the offers, the patients tended to judge an offer as less fair than did the normal controls, especially in the most unfair condition. This suggests that MDD patients may be more sensitive to fairness and are thus more likely to decide to reject unfair offers. This explanation is consistent with the speculation that a person’s sense of justice is a prerequisite for the rejection of unfair offers [49]. A second possible explanation is that the background emotional state may play an important role when making decisions. Previous studies have found that latent emotional states can alter people’s goals, attitudes, and risk perceptions, and some work, albeit limited, has been performed on how differently valenced emotions can influence social decision making [50,51]. Much empirical literature has illustrated the significant role of both task-related and induced emotion in decision-making behaviors in the ultimatum bargaining game [19,23,24]. Our study showed that the background negative emotional state seemed to alter the behavioral pattern of MDD patients in their social interactions, a finding that provides further evidence supporting the role of emotion in decision making.

A potential physiological mechanism for the decreased acceptance rate observed in MDD may be serotonin (5-HT) deficiency, which has been identified in MDD patients [27,28]. Recent studies have found that 5-HT plays a critical role in social decision-making behaviors and is a demonstrably powerful force shaping our social lives [25,26,49,52]. Crockett et al. observed that healthy volunteers were more likely to reject unfair offers after lowering their 5-HT levels with acute tryptophan depletion and less likely to reject unfair offers after enhancing 5-HT levels with citalopram [25,26]. Another study showed that individuals with a low level of serotonin transportation in the dorsal raphe nucleus were more likely to be honest and trustful, could not tolerate unfair offers, and thus were more likely to engage in personally costly forms of retaliation [49]. In addition, structural and functional dysfunction in the brain regions that play an important role in ultimatum bargaining behavior could be a neural basis for the decreased acceptance rate in MDD patients. Neuroimaging studies have indicated that specific regions associated with deliberative processes, cognitive conflict and emotional processing, such as the insula, the dorsolateral prefrontal cortex, the anterior cingulate cortex and the amygdala [2,23,53,54], may underlie the neural basis of a responder’s decision behavior in the UG. All these regions have shown structural and/or functional abnormalities during depressive episodes [29,30]. Future studies need to clarify whether neural dysfunction causes the difference between the ultimatum bargaining behavior of people with MDD and that of normal controls.

Another important finding in the present study is that the MDD group showed no differences in acceptance rates between human and computer partners, unlike the normal controls who accepted the most unfair (20%-10% of the stake size) offers with a lower offer size at a significantly lower rate when they were proposed by human partners than when the same offers were proposed by computer partners. This indicates that MDD patients merely care about offer fairness regardless of whether the proposer was a computer or human and seem not to consider reciprocity. In a healthy population, more seems to be tolerated in terms of behavior and actions from agent actors (computers) than from human actors [55]. For the indiscriminate treatment of unfair proposals from human partners and computer partners in the MDD group, we proposed several possible explanations. First, this abnormal decision behavior we observed may be related to the disturbed affective cognition, which is a major characteristic of patients with MDD (for reviews, see [56-60]). Specifically, MDD patients tend to attend selectively to negative stimuli in their environment and to interpret neutral or ambiguous stimuli as negative or as less positive. This negative bias has been observed in facial emotion processing, memory and attention, and the social and moral emotion of patients with MDD (for a review see [59]). We speculate that the negative bias also exists in decision making in a social interaction context in MDD. It is possible that negative bias is activated by unfair proposals, causing negative cognition to be automatically induced. This negative cognition thus makes patients with MDD neglect the non-social information of the computer and focus on the inequality *per se*. Thereby, they may interpret proposals from computer partners as negatively as they do those from human partners. Second, impaired social cognition may also play a role in the indiscriminate treatment of unfair proposals from human partners and computer partners in the patients with MDD. Researchers have found that intact social cognition, especially theory of mind, plays a critical role in distinguishing between unfair proposals from computer partners and human partners [40]. Participants need to understand and respond to the thoughts and feelings of others in the human proposer condition, while they do not need to conjecture the intentions of computers in the computer proposer condition. Previous studies exploring the social cognition of MDD have shown that depressed subjects showed impaired ability to conjecture the intentions of others [61-63]. It is possible that the dysfunction in social cognition makes MDD patients treat the human proposer and computer proposer indiscriminately. Additionally, we noted that our results obtained in the patients with MDD were similar to those obtained in patients with a lesion in the ventromedial prefrontal cortex (vmPFC), who also did not distinguish unfair offers from human and computerized opponents [10]. Considering that vmPFC is a brain region that is necessary for valuing social information in social interaction and decision-making [10,64], Moretti et al. think that the ability to value social information is important for the discriminate treatment of human proposers and computer proposers [10]. Abnormal structure and function in the vmPFC have been consistently reported in patients with MDD [65-67]. Therefore, it is possible that the indiscriminate treatment of human and computer proposers in MDD is related to the impaired functions which should be served by the vmPFC, such as valuing social information during interactive decision making. These speculations also need to be tested in future studies.

### Strengths and limitations

A strength of this study was our experimental design. On the one hand, we included human and computer offers and found aberrant features of decision making in MDD patients when facing unfair proposals from human partners and computer partners, which has never been explored. On the other hand, we set up two types of offer size (high and low) and varied the stake size across trials, so we investigated the effects of fairness and offer size separately, which were usually mixed in previous studies.

Meanwhile, several issues should be addressed. First, our sample size was small. To explore how likely the difference in acceptance rates between the two groups and those between human and computer proposers in each group is reliable, we conducted Bayesian *t*-tests for acceptance rates in different conditions using a Bayes factor calculation [68]. Our results revealed that the alternative hypothesis of a group effect on acceptance rate was 2.0 times more likely to be true than the null hypothesis of no group effect. In addition, we found that the alternative hypothesis of a proposer effect on acceptance rate was 4.2 times more likely to be true than the null hypothesis of no proposer effect in the NC group, while the null hypothesis of no proposer effect on acceptance rate was 2.8 times more likely to be true than the alternative hypothesis of a proposer effect in the MDD group. These results provide additional evidence for the group difference in acceptance rates of UG and distinguishing between unfair proposals from computer partners and human partners in the NC group but not the MDD group. Although the Bayes factor calculation showed some statistical power to detect existing effects, our findings still need to be validated repeatedly by future studies with larger sample sizes. Second, it is a flaw that we did not collect the data about socio-economic status and average salary of the participants and did not explicitly assess the meanings of each stake size for participants. However, according to the average annual income and living standards of local people [69], the two stationary offer sizes we used, even though they seem to have little difference on the surface, may have different meanings for these participants. Our finding that the offer size affected the rejection rate of unfair proposals supports this possibility and suggests a magnitude effect of offer size. In order to reach a firm conclusion about the magnitude effect of offer size, future studies should collect the data about socio-economic status and average salary of the participants, explicitly assess the meanings of stake size for participants and/or increase the differences in magnitude between offer sizes. Third, all but one of the patients in our study were taking antidepressant medications, which may confound our results. Future studies with drug-naïve MDD patients may distinguish the effects of medication on ultimatum bargaining behaviors from the effects of disease *per se*. Another future direction could be to compare the ultimatum bargaining behaviors of MDD patients before and after antidepressant medication treatment. Fourth, depression is often accompanied by symptoms of anxiety, which also has an impact on ultimatum bargaining behaviors [70]. Future studies need to control the effect of anxiety on social decision-making. Finally, anhedonia and sadness are separate major characteristics of MDD; however, different pathophysiological mechanisms underlie these two symptoms [28,71]. Patients with different symptoms may show differences in their ultimatum bargaining behaviors. It has been demonstrated that sadness plays an important role in biasing decision making [19,23,24], while anhedonia is related to decreased reward sensitivity, which appears to underlie a failure to maximize potential monetary earnings [72]. Future studies should investigate the relationship between specific symptoms of MDD and ultimatum bargaining behaviors.

## Conclusions

In summary, our findings provide further evidence supporting the role of emotion in decision making. More importantly, our findings indicate that depressed patients show altered decision-making behavior in social interaction contexts. Our findings also indicate an influence of clinical symptoms on everyday decision making in MDD patients, an issue that has been neglected but is obviously important to their lives. Future studies should further investigate the possible mechanisms, such as perception of fairness and the neurobiological basis, behind patients’ impaired social decision making. While it remains to be seen whether these aberrant decision characteristics can be validated in large independent studies, the preliminary findings reported here suggest the possibility that abnormality in the social decision-making process could be a potential marker for MDD diagnosis and prognosis. Many psychiatric disorders are related to one or more abnormal decision-making processes [73,74]. Therefore, a better understanding of these decision-making processes will undoubtedly improve the diagnosis and treatment of mental illness, and in turn, studies about unusual or aberrant decision-making behavior in patients may further promote an understanding of normal decision-making behavior.

## Competing interests

The authors declare that they have no competing interests.

## Authors’ contributions

Conceived and designed the experiments: YZ, YW, SL. Performed the experiments: YW, PW, GWW, ZNL. Analyzed the data: YW, YZ. Wrote the paper: YW, YZ, SL. All authors read and approved the final manuscript.

## Pre-publication history

The pre-publication history for this paper can be accessed here:

http://www.biomedcentral.com/1471-244X/14/18/prepub
